# Intensity-Based Assessment of Hippocampal Segmentation Algorithms Using Paired Precontrast and Postcontrast MRI

**DOI:** 10.3390/bioengineering12030258

**Published:** 2025-03-04

**Authors:** Justin Cramer, Leslie Baxter, Harrison Lang, Jonathon Parker, Alicia Chen, Nicholas Matthees, Ichiro Ikuta, Yalin Wang, Yuxiang Zhou

**Affiliations:** 1 Department of Radiology, Mayo Clinic Arizona, 5711 E Mayo Blvd, Phoenix, AZ 85054, USA; cramer.justin@mayo.edu (J.C.);; 2 Department of Neurosurgery, Mayo Clinic Arizona, 5711 E Mayo Blvd, Phoenix, AZ 85054, USA; 3 School of Computing and Augmented Intelligence, Arizona State University, 699 S Mill Ave, Tempe, AZ 85281, USA

**Keywords:** hippocampal segmentation, Alzheimer’s dementia, mesial temporal sclerosis, MRI volumetrics, segmentation refinement

## Abstract

Hippocampal segmentation is essential in neuroimaging for evaluating conditions like Alzheimer’s dementia and mesial temporal sclerosis, where small volume changes can significantly impact normative percentiles. However, inaccurate segmentation is common due to the inclusion of non-hippocampal structures such as choroid plexus and cerebrospinal fluid (CSF), leading to volumetric overestimation and confounding of functional analyses. Current methods of assessment largely rely on virtual or manual ground truth labels, which can fail to capture these inaccuracies. To address this shortcoming, this study introduces a more direct voxel intensity-based method of segmentation assessment. Using paired precontrast and postcontrast T1-weighted MRIs, hippocampal segmentations were refined by adding marginal gray matter and removing marginal CSF and enhancement to determine a total required correction volume. Six segmentation algorithms—e2dhipseg, HippMapp3r, hippodeep, AssemblyNet, FastSurfer, and QuickNat—were implemented and compared. HippMapp3r and e2dhipseg, followed closely by hippodeep, exhibited the least total correction volumes, indicating superior accuracy. Dedicated hippocampal segmentation algorithms outperformed whole-brain methods.

## 1. Introduction

Hippocampal segmentation with volume measurement is one of the most important quantitative tasks in neuroimaging, long utilized for assessing Alzheimer’s dementia and mesial temporal sclerosis in epilepsy [1,2]. Precision is critical, as small changes in volume result in large normative percentile shifts. For example, consider the nomogram of left hippocampal volume for females from Nobis et al., where a 1 mL decrease in volume (approximately 20–25%) for a 65-year-old drops them from the 80th to the 5th percentile for age [3].

Meanwhile, the inclusion of non-hippocampal structures such as the choroid plexus, basal vein of Rosenthal, posterior cerebral arteries, and cerebrospinal fluid (CSF) in hippocampal segmentations is commonly encountered and difficult to avoid, even during “gold standard” manual segmentation [4,5]. This is problematic for several reasons. First, the inclusion of non-hippocampal structures leads to volume overestimation. Given that choroid plexus volume can actually be inversely related to hippocampal volume in Alzheimer’s dementia [6], this is particularly concerning as a source of volumetric error. Second, the inclusion of non-hippocampal structures confounds studies of hippocampal function such as diffusion, perfusion, and blood-oxygen-level-dependent (BOLD) analysis, given the independence of CSF and choroid plexus from brain function [7].

Recently, researchers analyzed six different automated hippocampal segmentation algorithms [8]. These included algorithms specifically designed to segment the hippocampus (e2dhipseg [9], HippMapp3r [10], and hippodeep [11]) and whole brain segmentation algorithms (AssemblyNet [12], FastSurfer [13], and QuickNat [14]). They determined algorithm performance by comparing it to a generated “virtual” ground truth segmentation based on a consensus method using a simultaneous truth and performance level estimation (STAPLE) algorithm [15]. This analysis found non-superiority between FastSurfer, QuickNat, and hippodeep based on various metrics.

Besides the above STAPLE method, the most common method of assessing segmentation is a comparison with manually segmented “ground truth” labels. For example, a recent study assessed nine hippocampal segmentation methods on three different datasets against manual ground truth segmentations [16]. It found that algorithms generally performed best on public datasets and worse on private datasets. FastSurfer and hippodeep were the top performers on the private dataset. However, manual segmentations are time-consuming and prone to error and variability, with the inclusion of choroid plexus previously noted as difficult to avoid [4]. For similar reasons, purely qualitative assessment of segmentations is prone to error and variance as well.

For the above methods of assessment, none actually quantify or assess the amount of non-hippocampal inclusion in the segmentations. They rely on error-prone virtual or manual ground truth segmentations and typically report Dice scores or similar measures of accuracy. Meanwhile, anecdotally, we have observed much greater segmentation accuracy when comparing dedicated segmentation algorithms (e2dhipseg, Hippmapper, and hippodeep) to FastSurfer segmentations, which is not entirely accounted for by previous results. Given this perceived lack of clarity on the true accuracy of segmentation, we sought a more direct assessment of segmentation accuracy that is independent of any manual ground truth labels.

To directly assess hippocampal segmentation accuracy, first consider the composition of the hippocampus. It is a gray matter structure, though with interposed white matter structures to include the alveus and fimbria, which become the fornix posteriorly, and white matter tracts between the hippocampus and amygdala anteriorly [17]. It also contains tiny internal vascularity and sometimes cysts. An accurate hippocampal segmentation contains only those structures.

In our experience, we have observed three common patterns of hippocampal segmentation error: exclusion of hippocampal gray matter, inclusion of surrounding CSF, and inclusion of adjacent enhancing structures such as choroid plexus and blood vessels. We propose a method of hippocampal segmentation analysis that quantitatively assesses the intensity values of each voxel to determine the extent of each error present and ranks each segmentation algorithm accordingly. This method utilizes a dataset composed of paired and coregistered noncontrast and postcontrast MR sequences. It uses noncontrast MR images to assess gray matter and CSF and postcontrast MR images to assess enhancing structures. This method provides a more direct analysis of hippocampal segmentation accuracy and quantifies the extent to which segmentations contain non-hippocampal structures.

## 2. Materials and Methods

To summarize, precontrast and postcontrast 3D T1 sequences obtained during the same MRI scanning session on the same patient were collected retrospectively. The hippocampi were segmented on the T1 precontrast sequence with six different algorithms. Then, the gray matter was added, and CSF and enhancement were subtracted from the margins of these segmentations, with correction volumes recorded and compared.

### 2.1. Data Collection and Preprocessing

MRI brain examinations containing both 3D noncontrast and postcontrast T1 sequences during the same MRI on the same patient were retrospectively sought without regard to indication. The institutional PACS database was queried from August 2013 to August 2023 using an internal tool with institution-specific Series Descriptions for the desired sequences. These were reviewed and excluded if the sequence was misnamed, there was excessive motion, or the hippocampus was absent or severely distorted, as assessed by a board-certified neuroradiologist. MRIs were performed on a variety of General Electric (GE, Boston, MA, USA) (Discovery MR750w 3 Tesla (T), Signa PET/MR 3T, Signa HDxt 1.5 T) and Siemens (Berlin, Germany) (Skyra 3T, Magnetom Vida 3T) scanners. The noncontrast and postcontrast T1 sequences were both acquired with the fast spoiled gradient echo (FSPGR) technique for GE and the magnetization-prepared rapid acquisition gradient echo (MPRAGE) technique for Siemens.

Preprocessing of these MRIs was first performed. MRIs were anonymized by conversion to NIfTI format with dcm2niix [18]. ANTsPy [19] (version 0.3.7) was used to perform N4 bias field correction on both the T1 and T1 postcontrast sequences. The T1 sequence was then registered to an MNI template using ANTsPy with a linear/rigid “Similarity” transform (scaling, rotation, translation) and otherwise default parameters to include mutual information metric. This step also resampled the images to 1.0 mm isovoxel using B-Spline interpolation. The T1 postcontrast sequence was then registered to the T1 sequence with the same rigid transform technique. Finally, a subtraction image between the noncontrast and postcontrast acquisitions was created to maximize the difference between enhancing and nonenhancing voxels. This was performed by subtracting the T1 noncontrast sequence from the T1 postcontrast sequence with NumPy [20], first normalizing the intensities from 0–1, subtracting the intensity values between the 2 sequences, and scaling the values by 1000 to get values ranging from −1000 to 1000.

### 2.2. Hippocampal Segmentation

The hippocampi were segmented utilizing six different segmentation algorithms implemented in Python 3.8.19. Additional implementation details are described in Appendix A.2. After segmentation, outlier hippocampal volumes falling outside the interquartile range were identified and visually inspected.

### 2.3. Analysis of Hippocampal Segmentations

The hippocampal segmentations were then analyzed on a voxel intensity basis. To summarize, the hippocampal segmentations for each method were assessed by adding marginal (at the edges of the segmentation) gray matter, subtracting marginal CSF, and subtracting marginal enhancement. The volume of correction for each material (CSF, gray matter, and enhancement) and total required correction were calculated as described further below.

First, a subset of segmentations was visually inspected for quality. The hippodeep segmentations at a threshold of 1.0 were excluded from analysis due to excessive fragmentation of the segmentation; a threshold value of 1.0 was simply too high to contain a meaningful hippocampal segmentation.

Next, the segmentations were refined through a process of adding gray matter, subtracting enhancement, and subtracting CSF to the margins or outsides of the segmentations.

The reason corrections were not performed throughout the entire segmentation is that the hippocampus contains internal enhancing vessels and CSF intensity cysts, which should be included in the segmentation. If corrections were performed throughout the entire segmentation, these structures would also be removed and artificially inflate volumetric correction, confounding statistical analysis. Finally, given that the hippocampus is a solid and smooth structure, most segmentation errors do indeed occur at the margins.

The mixture of gray matter, CSF, and enhancement was chosen for several reasons. First, based on visual assessment, those are the most common hippocampal segmentation errors. We also considered removing white matter, as periventricular white matter can be erroneously included in segmentations. However, CSF subtraction tended to remove those white matter inclusions also. Moreover, other white matter structures, such as the fimbria and proximal fornix, are part of the hippocampus and should not be removed. The second reason for choosing these structures was their ability to assess segmentations at multiple threshold values (two of the algorithms return segmentations as probability maps, not binary labels). At very high thresholds, the hippocampal segmentation would shrink to be too small. While that segmentation would include no erroneous vascular structures or CSF, it was undersegmenting the hippocampus itself. The addition of marginal gray matter was necessary to avoid bias towards high threshold segmentations.

Gray matter was first added to the margins of each segmentation to include hippocampus not included in the original segmentation, accounting for undersegmentation of hippocampus. First, a range for gray matter signal intensity was calculated for each study. This was performed by creating an aggregate right hippocampal segmentation of all voxels shared by all the segmentations, creating the smallest segmentation agreed upon by all algorithms. The presence of probability maps made this aggregate method particularly useful because hippocampal size shrunk with increasing probability. The mean and standard deviation signal intensities of this smallest label on the T1 sequence were considered to represent the range for gray matter. Next, to constrain the area of gray matter addition, an aggregate segmentation of any voxels from any segmentations was created, essentially creating the largest segmentation. This was performed to avoid adding adjacent gray matter structures like the parahippocampal gyrus and made the assumption that the segmentations were not extremely inaccurate, which was confirmed on initial visual inspection. Next, each hippocampal label was dilated one iteration using the SciPy binary_dilation method. Voxels were subtracted from this dilated shell if they fell outside the gray matter signal intensity range (mean ± 1 standard deviation) or if they fell outside the aggregate largest segmentation. Enhancing voxels, as determined below, were also subtracted from the shell to avoid adding isointense structures such as the choroid plexus. Finally, the added voxels were joined to the original segmentation, and any islands (stray voxels not connected to the hippocampus) were removed. The volume of added gray matter was recorded.

CSF was then removed from the margins of each segmentation. First, a range for CSF signal intensity was calculated for each study. This was performed by extracting the right lateral ventricle voxels from the FastSurfer whole brain segmentation. Enhancing indices, calculated as described below, were subtracted from the right lateral ventricle mask to exclude the choroid plexus. The mean and standard deviation signal intensities of this right lateral ventricle mask on the T1 sequences were considered to represent the intensity range for CSF. Next, an aggregate shell was created from all the segmentations, consisting of all voxels not shared by all segmentations. The margin for CSF subtraction was the overlap between that aggregate shell and the individual segmentation. This method was used instead of just doing a shrink operation on each segmentation to create a shell because it allowed for a complete correction of volumes. For example, consider e2dhipseg at a threshold of 0, which would be a relatively large segmentation. It could include CSF pretty extensively, and shrinking it by one voxel would not capture all the erroneous CSF. CSF intensity was then subtracted from that margin, and islands were removed. The volume of subtracted CSF was recorded.

**Figure 1:**
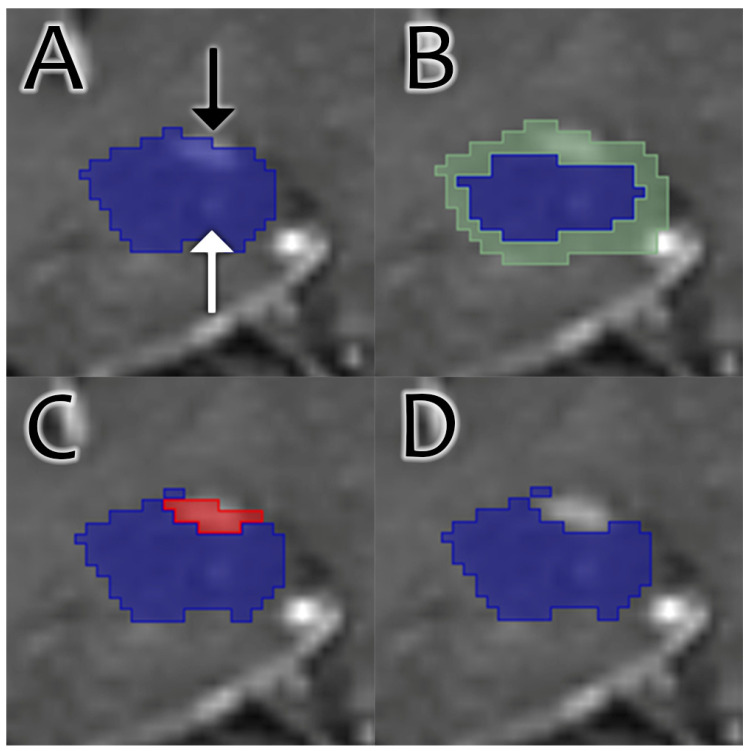
Coronal images through the hippocampus on a T1 postcontrast sequence demonstrate the process of contrast removal. (**A**) The original segmentation (blue). Erroneous inclusion of choroid plexus is apparent (black arrow), as is the correct inclusion of a faintly visible intrahippocampal vessel (white arrow). (**B**) An aggregate shell (green) is generated around the original segmentation. (**C**) Enhancing voxels (red) are identified within the shell. The choroid plexus enhancement is identified, while the intrahippocampal enhancement falling outside the shell is not. (**D**) Final result. The enhancing choroid plexus is removed, while intrahippocampal enhancement is preserved.

Enhancement was then removed from the margins of the segmentation. This process is illustrated in Figure 1. First, a range for enhancement intensity was calculated for each study. This was performed by calculating the mean and standard deviation of the subtraction image created, as described in Section 2.1. Enhancement was then subtracted from the same margin calculated for CSF subtraction and was based on signal intensity on the subtraction image. Islands were then removed. The volume of enhancement was recorded.

The total correction volume for each segmentation was calculated as the sum of gray matter added, CSF removed, and enhancement removed.

### 2.4. Statistics

Results were analyzed to determine the hippocampal segmentations requiring the least total volume correction and to analyze statistical differences between segmentations. Statistical analysis was performed in Python utilizing the NumPy and SciPy libraries.

First, mean correction volumes were calculated for each segmentation algorithm. Mean volumes were calculated for each substance (gray matter, CSF, and enhancement) as well as total correction volume (defined as gray matter added + CSF removed + enhancement removed). The top-performing (least correction required) thresholds for hippodeep and e2dhipseg were selected for further analysis along with the other four algorithms.

Next, a Shapiro–Wilk normality test was run on the total correction for each algorithm and determined a non-normal distribution of data in each case. Then, Levene’s test was conducted with a result of 0, indicating heterogeneity of variances, so Welch’s ANOVA test was selected to assess for significant differences between the groups. The *p*-value for Welch’s ANOVA test was 0, indicating significant differences were present. Finally, a post hoc Tukey’s Honestly Significant Difference (HSD) test was performed to assess for significant differences between the groups.

## 3. Results

### 3.1. Data Collection and Preprocessing

A total of 266 examinations were identified by the initial query. After review, 24 were excluded for a total of 242 MRIs. Reasons for exclusion included distortion of the hippocampus (14), excessive motion (5), and incorrectly labeled sequences (5). The intravenous contrast utilized was gadobutrol. Voxel dimensions ranged from 0.377 to 1.0 mm (though they were all resampled to 1.0 mm using B-spline interpolation). Demographic information is summarized in Table 1.

### 3.2. Hippocampal Segmentation

The algorithms were largely successful in segmenting the hippocampi. Failures identified by visual inspection of outliers are summarized in Table 2. HippMapp3r failed three times, e2dhipseg failed four times, QuickNat failed six times, and the other three algorithms had no failures. Every failure was on FSPGR sequences performed on a GE Discovery MR750w MRI. Visually, these sequences demonstrated relatively less gray-white differentiation/contrast, potentially accounting for the failures.

### 3.3. Refinement of Hippocampal Segmentations

**Figure 2:**
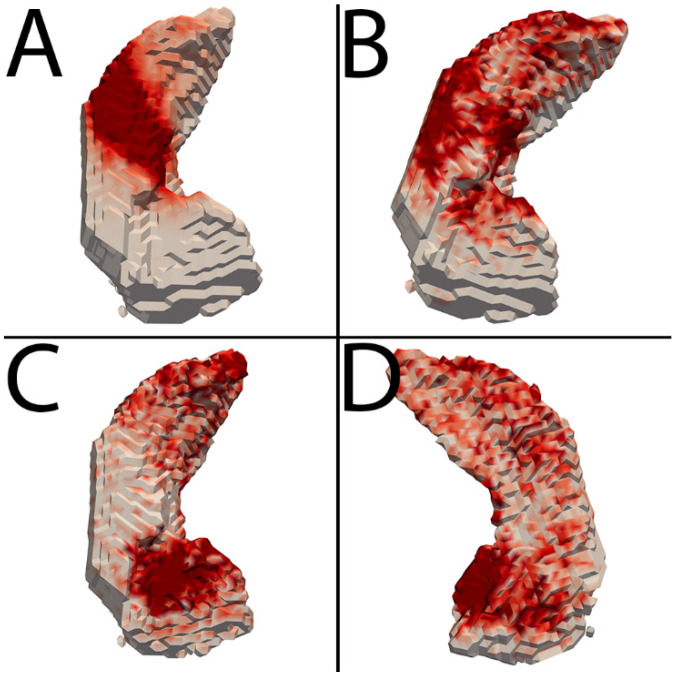
Heatmaps depicting average refinement results. The hippocampus is depicted in white, with progressively darker red regions representing more common areas of refinement. (**A**–**C**) are a top-down view of the hippocampus, with the anterior portion at the bottom. (**D**) is a bottom-up view with the anterior portion at the bottom. (**A**) Enhancement removal. This was most common along the superior aspect of the hippocampus at the expected location of the choroid plexus. (**B**) CSF removal. This was most common along the superior aspect of the hippocampus also, with relatively less removal towards the center at choroid plexus. (**C**,**D**) Gray matter addition. Addition occurred most frequently at the anterosuperior and anteromedial margins of the hippocampus, with a fairly diffuse lesser distribution throughout the hippocampus.

Heatmaps in Figure 2 demonstrate the overall spatial distribution of refinement. In general, the choroid plexus represented the majority of enhancement removal, and CSF along the superior hippocampus around the choroid plexus represented the majority of CSF removal. Gray matter addition, a smaller overall contributor, occurred diffusely throughout the hippocampus and slightly more at the anterior and posterior poles.

Quantitative results of hippocampal refinement operations are summarized in Table 3, which conveys the original hippocampal volumes, volume of gray matter (GM) added, CSF removed, enhancement removed, and total volume of correction (GM + CSF + enhancement) for each algorithm. Table 3 includes only the top-performing thresholds of e2dhipseg (0.5) and hippodeep (0.3). Table A1 is provided in the Appendix A for reference and completeness and includes all the thresholds for e2dhipseg and hippodeep. These tables demonstrate that the algorithm requiring the least total average correction (best performance) was HippMapp3r (0.45 mL), followed by e2dhipseg at a threshold of 0.5 (0.51 mL) and hippodeep at a threshold of 0.3 (0.56 mL). The whole brain algorithms required more correction, with AssemblyNet requiring 0.83 mL, FastSurfer 0.94 mL, and QuickNat 1.14 mL.

Some other trends are apparent in Table 3. First, the amount of gray matter added was relatively consistent, varying by at most 0.15 mL. The amount of CSF removed varied by up to 0.26 mL, and the amount of enhancement removed varied by up to 0.50 mL. In other words, CSF and enhancement removal were larger differentiators, while gray matter addition largely served to penalize undersegmentation among higher thresholds.

**Figure 3:**
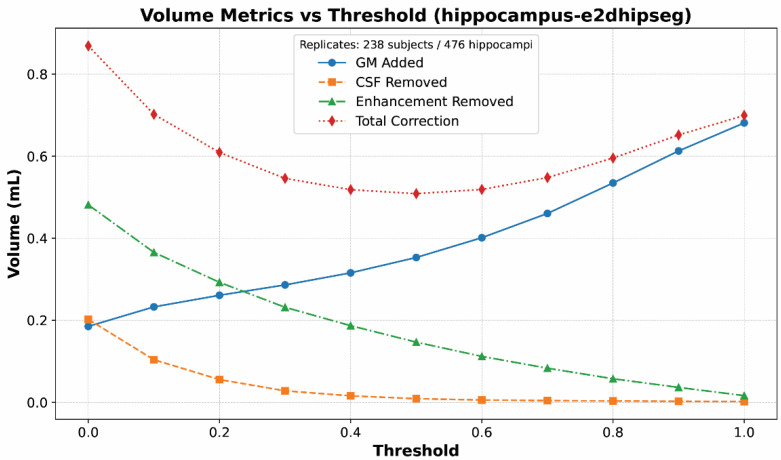
Line graph of mean volume corrections for each substance (gray matter, CSF, enhancement) at each threshold value for e2dhipseg. This graph demonstrates the expected trends: With increasing threshold values, the segmentation becomes smaller, more gray matter is excluded, and less CSF and enhancement are included. An optimal cutoff of 0.5 can be seen as the lowest point for the total correction values.

Another trend apparent from Table 3 is that the refinement had the intended effects on thresholded segmentations. This is illustrated more clearly in Figure 3, which shows volume corrections at different e2dhipseg thresholds. As expected, with increasing thresholds, the segmentations included less CSF and enhancement but excluded more actual hippocampal gray matter because the segmentations were smaller. The optimum threshold minimized the total required correction.

**Figure 4:**
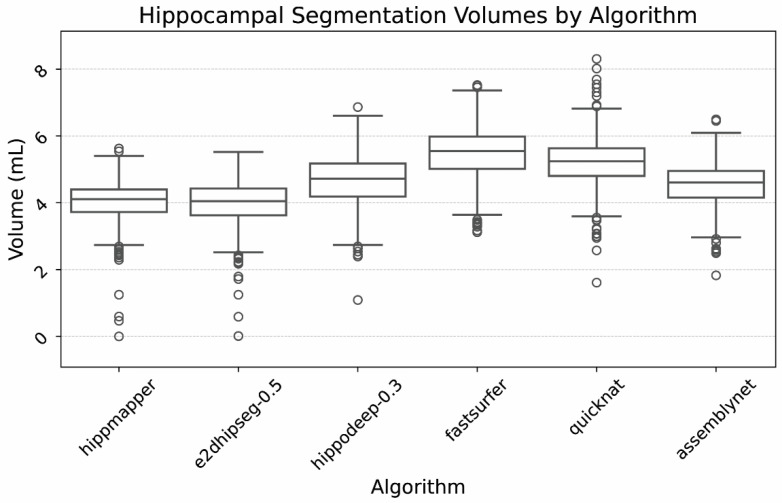
Box plot of hippocampal volumes by algorithm. Outliers were due to severe hippocampal volume loss as well as segmentation failures, as confirmed by visual inspection.

Average hippocampal volumes also varied by algorithm, which is depicted in Figure 4. Mean volumes for hippocampi ranged from 4.0 to 5.5 mL, depending on the algorithm and threshold used.

**Figure 5:**
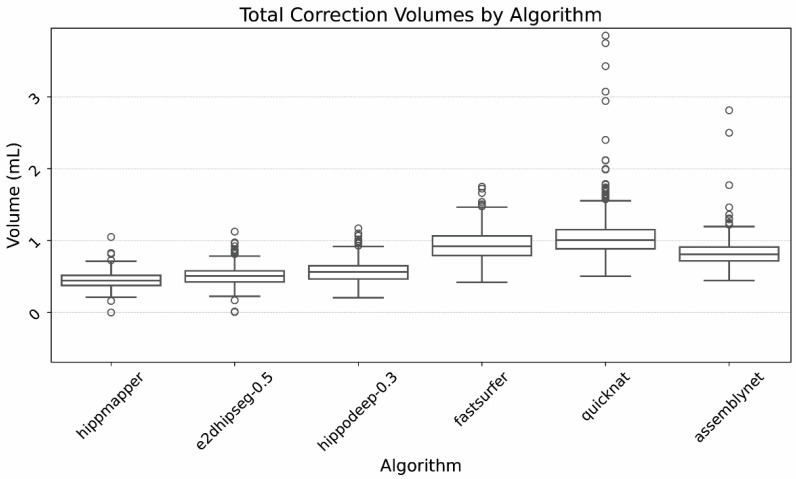
Box plot of hippocampal correction volumes, defined as the amount of gray matter added, CSF subtracted, and enhancement subtracted per hippocampus. Outliers largely related to large segmentation errors that were excluded by island removal in the refinement process.

Finally, Figure 5 illustrates the ranges of total required correction volumes. Again, demonstrated are relatively similar correction volumes between the dedicated hippocampal segmentation algorithms, and larger correction volumes for the whole brain algorithms.

### 3.4. Statistics

The results of the statistical analysis are summarized in Table 4. Post hoc Tukey’s HSD test performed on the total correction volume showed no significant difference between the two top-performing algorithms, HippMapp3r and e2dhipseg, at a threshold of 0.5. It also found no significant difference between the second and third performing algorithms, e2dhipseg at 0.5 and hippodeep at a threshold of 0.3, but did find a difference between the first and third, HippMapp3r and hippodeep. All other algorithms were found to be significantly different in terms of correction volumes required.

## 4. Discussion

This study describes a direct assessment of six hippocampal segmentation algorithms based on voxel intensities. It found HippMapp3r and e2dhipseg (0.5 threshold) to be superior and equivalent in terms of total required correction volumes, followed closely by hippodeep (0.3 threshold). Dedicated hippocampal segmentation algorithms outperformed whole brain segmentations, requiring 0.5–0.6 mL of correction compared with 0.8–1.1 mL for the whole brain algorithms. These are clinically significant volumes, with a difference of 1 mL shifting patients from the 80th to the 5th percentile for age-normalized volume [3].

Our analysis method utilized a dataset of both precontrast and postcontrast T1-weighted images. To be clear, segmentations were only performed on T1 precontrast images, and the postcontrast images were only used for analysis.

This method of assessment was born out of a perceived deficiency in existing knowledge about hippocampal segmentation accuracy. Prior assessments rely on manually segmented ground truth or aggregate virtual ground truth labels and do not explicitly quantify the extent to which a segmentation contains only the hippocampus. Moreover, manual segmentations of the hippocampus are prone to error, further confounding such analysis. Precise segmentation of only hippocampal gray and white matter is important not just for volumetric determination but also for studies of hippocampal function, as erroneous inclusion of CSF and vascular structures can significantly alter the results of functional studies.

Our results differ from recent prior publications [8,16], which assessed algorithms against manual or virtual ground truth labels. One study found no single outperforming algorithm, with FastSurfer performing best in VS, QuickNat in DICE and average HD, and hippodeep in HD [8]. This paper also tested hippodeep at one threshold level (0.5). Another paper found similar performance between hippodeep and Fastsurfer on a private dataset [16] and different results on public datasets. In comparison, our study utilized a private dataset, assessed hippodeep and e2dhipseg at 11 different threshold levels, and found HippMapp3r and e2dhipseg at a threshold of 0.5 to be superior and equivalent, followed closely by hippodeep.

Our intensity-based method has several advantages. First, it objectively assesses the accuracy of hippocampal segmentation versus traditional virtual and manual ground truth methods by eliminating the variability of manual segmentation. Second, by not requiring manual segmentations for comparison, it allows for the selection of potentially larger and different datasets. In the case of this study, it allowed for the curation of a unique internal dataset of precontrast and postcontrast paired images. An important limitation is the requirement of postcontrast sequences, which are not widely available in public datasets. Finally, a version of this method could also be used to create a new hippocampal segmentation algorithm by refining existing labels and then retraining a neural network.

Despite our findings of two top-performing algorithms, the data provided in Table A1 can serve as a reference to inform different research priorities. For example, a researcher wanting to assess hippocampal function could utilize e2hipseg at a threshold of 0.8 and be reassured they are including a large portion of the hippocampus and nearly no CSF or choroid plexus, even if the hippocampal volumes may be slightly underestimated.

We observed 13 failures occurring between HippMapp3r, e2dhipseg, and QuickNat. These all occurred on the same scanner and sequence, which visually contained less gray-white contrast and could account for the failures. It is important to note that the decreased contrast could be due to local scan parameters, and is not intended as a general assessment of that scanner. Given that HippMapp3r and e2dhipseg were the top-performing algorithms, some vigilance for failure detection, for example outlier review, is warranted if implementing these algorithms.

We found that CSF and enhancement removal were the main drivers of rankings, while gray matter addition largely served to filter out undersegmentation among higher thresholds. This makes sense based on a qualitative review of segmentations. Undersegmentation of the hippocampi is rare and minimal, while erroneous inclusion of CSF and enhancing structures is very common and extensive and served as the impetus for this study. The heatmaps in Figure 1 illustrate that the superior aspect of the hippocampus is the main source of error, representing the majority of enhancement and CSF removal.

This study has several limitations and caveats. First, this intensity-based method assumes relatively accurate segmentations at baseline. It is agnostic to the hippocampus location and performs refinement only at the margins for statistical considerations, as mentioned earlier. It is a suitable method for comparing and assessing segmentations but it would need to be coupled with ground truth and more traditional measures like DICE scores for new segmentations.

Second, the hippocampus is the most popular segmentation target in the brain, and newer segmentation algorithms are always becoming available. This is not a comprehensive assessment of all algorithms. However, this technique could be easily applied to additional algorithms. This method was not intended to produce perfect segmentations, only to assess and compare them. Thus, the required correction volumes are estimates for the purposes of comparison, and should not be used as a correction factor for any given algorithm.

As highlighted in our review of other recent hippocampal segmentation analyses and segmentation failures on this project, performance can vary based on datasets. A relative strength of this study is the use of real-world clinical data, though it is still only evaluating performance on a small subset of GE and Siemens MRIs. While these results may guide algorithm selection, monitoring and visually assessing segmentation performance remains essential.

Finally, we cannot definitively conclude that our results are more correct than those based on comparison with manual ground truth, just that they were different. Further validation would require correlation with clinical metrics and is a direction for future investigation.

## 5. Conclusions

Direct voxel intensity-based assessment of hippocampal segmentation algorithms shows that HippMapp3r and e2dhipseg perform the most accurate segmentations, followed closely by hippodeep. Dedicated algorithms generally performed better than whole-brain algorithms at hippocampal segmentation. This new method of segmentation assessment circumvents manual ground truth labels and quantifies the extent and type of error for each segmentation method, which may help guide algorithm selection.

## Figures and Tables

**Table 1:** Demographics.

Demographic	Result
Number of patients	242
Sex (female)	50%
Patient age (years)	
Range	3–91
Mean	49

**Table 2:** Segmentation failures.

Algorithm	Failures
HippMapp3r	3
e2dhipseg-0.5	4
hippodeep-0.3	0
AssemblyNet	0
FastSurfer	0
QuickNat	6

**Table 3:** Mean hippocampal volumes and correction volumes.

Algorithm	Total Correction (mL)	Original Volume (mL)	GM Added (mL)	CSF Removed (mL)	Enhancement Removed (mL)
HippMapp3r	0.446	4.026	0.337	0.007	0.103
e2dhipseg-0.5	0.509	3.960	0.353	0.009	0.147
hippodeep-0.3	0.564	4.646	0.266	0.025	0.274
AssemblyNet	0.834	4.553	0.336	0.110	0.387
FastSurfer	0.939	5.521	0.201	0.160	0.578
QuickNat	1.141	5.274	0.299	0.263	0.579

**Table 4:** Comparison of mean total corrections—Tukey HSD.

Group 1	Group 2	Mean Difference	*p*	Lower	Upper	Significant Difference (*p* < 0.05)
e2dhipseg-0.5	HippMapp3r	−0.072	0.207	−0.162	0.019	No
e2dhipseg-0.5	hippodeep-0.3	0.056	0.492	−0.035	0.146	No
AssemblyNet	e2dhipseg-0.5	−0.325	0.000	−0.416	−0.235	Yes
AssemblyNet	FastSurfer	0.139	0.000	0.049	0.229	Yes
AssemblyNet	HippMapp3r	−0.397	0.000	−0.488	−0.307	Yes
AssemblyNet	hippodeep-0.3	−0.270	0.000	−0.360	−0.179	Yes
AssemblyNet	QuickNat	0.307	0.000	0.217	0.398	Yes
e2dhipseg-0.5	FastSurfer	0.464	0.000	0.374	0.555	Yes
e2dhipseg-0.5	QuickNat	0.633	0.000	0.542	0.723	Yes
FastSurfer	HippMapp3r	−0.536	0.000	−0.627	−0.446	Yes
FastSurfer	hippodeep-0.3	−0.409	0.000	−0.499	−0.318	Yes
FastSurfer	QuickNat	0.168	0.000	0.078	0.259	Yes
HippMapp3r	hippodeep-0.3	0.128	0.001	0.037	0.218	Yes
HippMapp3r	QuickNat	0.705	0.000	0.614	0.795	Yes
hippodeep-0.3	QuickNat	0.577	0.000	0.486	0.667	Yes

Statistical analysis of differences between mean total correction volumes for segmentation algorithms. Top-performing thresholds for e2dhipseg and hippodeep were selected. There was no significant difference between e2dhipseg at a threshold of 0.5 and HippMapp3r, and e2dhipseg-0.5 and hippodeep at a threshold of 0.3.

## Data Availability

Data used for analysis are proprietary and cannot be shared publicly. The code utilized for segmentation and analysis is shared per the URL provided in Supplementary Materials.

## Supplementary Materials

The code created for performing and analyzing hippocampal segmentations in the fashion described is provided at https://github.com/radiplab/hippo-seg-analysis (Last updated 22 November 2024).

## Abbreviations

The following abbreviations are used in this manuscript:

AD	Alzheimer’s dementia
BOLD	Blood-oxygen-level-dependent
HD	Hausdorff distance
MNI	Montreal Neurological Institute
RAS	Right-anterior-superior
STAPLE	Simultaneous truth and performance level estimation
VS	Volumetric Similarity

## Appendix A

### Appendix A.1

A more complete version of Table 3 is included for completeness and reference below. Mean hippocampal volumes and correction volumes are included for every threshold value of e2dhipseg and hippodeep.

**Table A1:** Mean volumes sorted by total volume correction.

Algorithm	Original Hippocampal Volume (mL)	GM Added (mL)	CSF Removed (mL)	Enhancement Removed (mL)	Total Volume Correction (mL)
Hippmapper	4.026	0.337	0.007	0.103	0.446
e2dhipseg-0.5	3.960	0.353	0.009	0.147	0.509
e2dhipseg-0.4	4.221	0.316	0.016	0.187	0.518
e2dhipseg-0.6	3.706	0.401	0.006	0.112	0.519
e2dhipseg-0.3	4.492	0.286	0.028	0.232	0.546
e2dhipseg-0.7	3.457	0.460	0.004	0.083	0.548
hippodeep-0.3	4.646	0.266	0.025	0.274	0.564
hippodeep-0.4	4.381	0.313	0.017	0.234	0.565
hippodeep-0.5	4.126	0.370	0.013	0.199	0.582
hippodeep-0.2	4.963	0.220	0.039	0.324	0.583
e2dhipseg-0.8	3.183	0.535	0.003	0.057	0.596
e2dhipseg-0.2	4.817	0.261	0.056	0.293	0.609
hippodeep-0.6	3.885	0.434	0.010	0.167	0.611
hippodeep-0.1	5.248	0.185	0.058	0.371	0.614
hippodeep-1e-06	5.248	0.185	0.058	0.371	0.614
e2dhipseg-0.9	2.881	0.613	0.003	0.036	0.652
hippodeep-0.7	3.630	0.507	0.008	0.138	0.653
e2dhipseg-1.0	2.444	0.681	0.002	0.016	0.699
e2dhipseg-0.1	5.195	0.233	0.104	0.366	0.702
hippodeep-0.8	3.359	0.588	0.006	0.109	0.703
hippodeep-0.9	3.000	0.682	0.005	0.076	0.762
AssemblyNet	4.553	0.336	0.110	0.387	0.834
e2dhipseg-1e-06	5.783	0.185	0.202	0.482	0.869
FastSurfer	5.521	0.201	0.160	0.578	0.939
QuickNat	5.274	0.299	0.263	0.579	1.141

Mean corrections required for each hippocampal segmentation, grouped by material (gray matter, CSF, enhancement) and sorted by total required correction. All threshold values are included in this table for completeness and reference. Total correction is gray matter added + CSF removed + enhancement removed, with the lowest number indicating the best segmentation (least required correction) by this analysis. Correlate with Figure 3, which illustrates trends from this table in correction volumes by material and threshold value for e2dhipseg.

### Appendix A.2

Six different hippocampal segmentation algorithms were implemented in Python. The following describes some helpful implementation details.

e2dhipseg returned a probability map including both hippocampi with values ranging from zero to one. The hippocampi were separated by assessing the x coordinate of each label’s centroid and dividing them into right and left labels. Then, 11 different labels were saved for each hippocampus at progressive probability threshold levels (>0, >0.1 … >0.9, 1). In general, the hippocampal segmentation grew smaller with increasing probability. This was performed to assess the optimal threshold level for e2dhipseg.

HippMapp3r required additional preprocessing of brain extraction and conversion to right-anterior-superior (RAS) orientation. Brain extraction was performed by registering each T1 image to MNI, then transforming the MNI brain mask back to the native space using the inverse transform. Conversion to RAS orientation was performed using the FreeSurfer mri_convert command. HippMapp3r returned a direct label map of the hippocampi.

hippodeep returned probability maps of each hippocampus. As with e2dhipseg, 11 different labels of each hippocampus were saved at progressive probability levels.

AssemblyNet, FastSurfer, and QuickNat all performed whole brain segmentation. The hippocampal labels were extracted from these segmentations and saved separately. QuickNat required running the FreeSurfer mri_convert—conform command as a preprocessing step.
